# What factors are associated with physical therapists’ use of patient-reported outcome measures in managing patients with low back pain in primary health care in Sweden?

**DOI:** 10.1016/j.bjpt.2025.101250

**Published:** 2025-08-12

**Authors:** Christine Melbye, Sara Östhols, Philip von Rosen, Eva Rasmussen-Barr

**Affiliations:** Karolinska Institutet, Department of Neurobiology, Care Sciences and Society, Division of Physical therapy, Alfred Nobels Allé 23, 141 83 Huddinge, Stockholm Sweden

**Keywords:** Bio-psychosocial, Physical therapy, Rehabilitation, Examination, Clinical decision-making, PROM

## Abstract

**Background:**

Physical therapists' use of patient-reported outcome measures (PROMs) in managing patients with low back pain (LBP) is reportedly low, especially for health-related and psychosocial factors.

**Objective:**

To investigate factors associated with using specific PROMs among physical therapists working in primary care.

**Methods:**

We analyzed data collected from physical therapists (*n* = 1237). Logistic regression analyses (Odd Ratios (OR), 95 % Confidence Intervals) were conducted to investigate how using PROMs for pain, disability, health-related, and psychosocial factors is associated with the physical therapists' demographic characteristics, including educational levels.

**Results:**

Most physical therapists used PROMs for pain (83 %), while PROMs for disability (28 %), health-related (14 %), and psychosocial factors (13 %) were used less frequently. Being female (OR 2.57, 95 % CI: 1.84, 3.59) and working in private clinics (OR 1.83, 95 % CI: 1.27, 2.67) were associated with using PROMs for pain. Holding a master’s degree or PhD was linked to using PROMs for disability (OR 1.85, 95 % CI: 1.28, 2.66) and psychosocial factors (OR 2.11, 95 % CI: 1.19, 3.65). Being female (OR 1.51, 95 % CI 1.01, 2.29) and being an advanced clinical specialist (OR 2.09, 95 % CI: 1.29, 3.33) were associated with using PROMs for health-related factors.

**Conclusions:**

While physical therapists commonly use PROMs for pain, few use them to assess health-related and psychosocial factors. Those with higher educational levels or advanced clinical specialists are more likely to incorporate such PROMs in managing patients with LBP. Future studies should explore educational interventions to improve understanding of PROMs and their role in clinical reasoning within a biopsychosocial framework.

## Highlights

• Few physical therapists use PROMs for health-related and psychosocial factors.
• Higher education levels increase the likelihood of using these PROMs.
• Being an advanced clinical specialist increases the likelihood of using these PROMs.
• Understanding PROMs within the biopsychosocial framework is essential.

## Introduction

Low back pain (LBP) continues to be the leading cause of years lived with disability, resulting not only in suffering for the individual but also in significant economic impacts on society.^1^^,^^2^ Most individuals with LBP experience non-specific pain, which has a variable prognosis and is defined as not being attributable to a known specific pathology.^3^

Patients suffering from LBP are frequently seen in primary healthcare settings.^4^ In Sweden, as in many other countries, patients have direct access to or the option of self-referral to a physical therapist, potentially making the physical therapist the first healthcare professional a patient encounters. This places a considerable responsibility on the physical therapist to manage the patient's condition.^5^ This responsibility involves navigating uncertainties and clinical risks related to identifying serious pathologies while considering modifiable prognostic factors through a biopsychosocial model.^6^^,^^7^ Clinical guidelines recommend incorporating health-related and psychosocial factors into clinical reasoning.^8^, ^9^, ^10^ Thus, early identification of patients at risk for a poor prognosis addressing modifiable factors within the rehabilitation process is crucial for achieving a successful outcome.^11^^,^^12^ Utilizing patient-reported outcome measures (PROMs) to screen for factors in patient management is recommended. This approach enhances clinical examination, promoting shared decision-making and patient-centered care.^13^, ^14^, ^15^, ^16^, ^17^ This has also been highlighted by the World Health Organization (WHO).^18^ PROMs are “*any report of the status of a patient’s health condition that comes directly from the patient, without interpretation of the patient’s response by a clinician or anyone else*”.^19^

Numerous PROMs measuring various factors and constructs are available and recommended.^20^, ^21^, ^22^ To target treatment, guidelines recommend shorter PROMs, such as the STarT Back tool, which includes questions on several constructs to stratify patients according to specific risk levels based on prognostic factors.^23^^,^^24^ The outcome from the STarT Back tool is shown to be positively related to factors important for recovery, such as kinesiophobia, pain catastrophizing, disability, and pain intensity.^25^

Previous research has yielded mixed results regarding the use of PROMs by physical therapists managing patients with LBP in primary healthcare related to health and psychosocial factors.^17^^,^^26^^,^^27^ One study indicated that physical therapists in primary health care in Sweden used PROMs for pain and disability at a higher rate (>40 %), while their usage of PROMs for health-related and psychosocial factors was much lower (<15 %).^26^ A similar trend was observed among physical therapists in Spain, who assessed psychosocial factors to a very low extent and employed structured PROMs for these factors even less frequently.^28^ In addition, research has shown conflicting results regarding the physical therapists’ ability to identify psychosocial factors.^29^, ^30^, ^31^

Research has identified several barriers that hinder physical therapists' use of PROMs in their clinical practice. These barriers include the resources, time, and competence required for the use of appropriate PROMs.^26^, ^27^, ^28^^,^^32^, ^33^, ^34^, ^35^, ^36^, ^37^, ^38^, ^39^, ^40^, ^41^ While some studies suggest that knowledge about PROMs or having higher education may significantly affect usage, other studies have found no such association.^28^^,^^29^^,^^40^^,^^42^ There is a paucity of studies examining factors that influence or impact physical therapists' use of specific PROMs for health-related and psychosocial factors. To enhance physical therapists' understanding of and encourage the use of PROMs, particularly those addressing health-related and psychosocial aspects, it is essential to gain more insight into the factors influencing their use. Therefore, we aimed to investigate the factors affecting the use of PROMs among physical therapists managing patients with LBP within a Swedish primary health context, regarding PROMs for pain, disability, health-related, and psychosocial factors.

## Methods

### Design

This study used survey data collected from physical therapists working in primary health care in Sweden as part of a previously published study.^26^ Details and results of the study are presented elsewhere.^26^ Ethical approval for the study was obtained from the Regional Ethical Committee in Stockholm.

### Context of the study

Physical therapy is a regulated profession in Sweden with a protected title and requires a license to practice. There are around 15,000 registered physical therapists in Sweden.^43^ In 2015, 76 % of all physical therapists in Sweden were registered with the Swedish Association of Physical Therapists, and 77 % were female. In Sweden, physical therapy education (three years) leads to a Degree of Bachelor of Science (BSc) in Physical therapy. Physical therapists with a BSc in physical therapy are qualified to enter a master’s program, which in Sweden has a duration of one or two years. The advanced specialist level includes a master’s level 1 and supervised clinical work within the specialist area for at least three years. Approximately 750 physical therapists in Sweden are at an advanced physical therapy level. All patients in Sweden have access to and may utilize publicly financed primary health care with direct access to physical therapy treatment at a reasonable cost.

### Participants and process

A web-based survey (SurveyMonkey®) was mailed to 4934 physical therapists in Sweden to collect data on the use of PROMs and clinical tests by physical therapists when managing patients with LBP in primary health care. E-mail addresses were obtained from the Swedish Association of Physical therapists’ subdivisions of manual therapy, primary health care, pain management, and sports medicine. Members of the chosen subdivisions of the Swedish Physical Therapy Association were considered likely to meet and manage patients with LBP seeking primary care. Eligibility to participate in the study included working as a physical therapist in primary health care and regularly managing patients seeking care for LBP. Participants who answered that they did not work in primary health care or did not manage patients with LBP were excluded. The study included 1237 (25 %) participants who answered the survey and gave their informed consent to participate in the survey.

#### Demographic and educational factors

The survey comprised questions on demographic factors including educational level: sex (man/woman), education level (up to Bachelor level/Master and higher), advanced clinical specialist (yes/no), further education towards specialist (yes/no), current working setting (private clinic/public health care centre), years of working experience (<5 /6–10/11–15/ 16–20/>20), how often meeting patients with LBP (daily/1–4 times per week/less than once /week).

#### Outcome

The choice of the PROMs included in the survey was based on available PROMs in the Swedish clinical context and on dialogue with researchers and physical therapists in primary health care. The PROMs were classified into four groups.

• Pain (*Numeric Pain Rating Scale,**^44^* *Visual Analog Scale,**^45^* *Borg’s Category Scale**^46^*)
• Disability (*Oswestry Disability Index,**^47^* *Roland-Morris Disability Questionnaire,**^48^* *Pain Disability Index,**^49^* *Disability Rating Index,**^50^* *Patient-Specific Functioning Scale*^51^).
• Health-related (*Short Form 36,**^52^* *Euroquol 5 dimensions**^53^*).
• Psychosocial factors, including fear-avoidance /kinesiophobia (*Tampa Scale of Kinesiophobia,**^54^* *Fear Avoidance Beliefs Questionnaire*^55^), mental health (*Montgomery Åsberg Depression Rating Scale,**^56^* *Hamilton Anxiety Rating Scale,**^57^* *Beck's Depression Inventory**^58^*), self-efficacy (*Self-efficacy Scale**^59^*) and stratifying for risk (*Örebro Musculoskeletal Pain Questionnaire*,*^60^* *the Start Back Tool**^24^*)*.*

The participants answered multiple-choice questions concerning how frequently they used the specific PROMs. Each question was answered on a 6-point Likert scale (*“always”, “very often”, “often”, “seldom”, “very seldom”, or “never”).* The answers were then dichotomized into two categories: *“always, very often, often”* and *“seldom, very seldom, never".* If a participant marked *“always” “very often”* or *“often”* for at least one of the PROMs included in the specific PROM group, the participant was classified as if using PROMs in that specific PROM group. Only one answer was possible for each PROM.

### Statistical analysis

Participants' demographics and frequency response data were expressed using descriptive statistics. Logistic regression analyses were conducted to analyse the physical therapists’ use of PROMs, with separate models for each PROM group: Pain, Disability, Quality of Life, and Psychosocial factors.

First, univariable analyses were performed to explore the associations between the independent variables and each PROM group. Independent variables with a p-value < 0.20 in the univariable regression analyses were included in the backward logistic regression analyses and retained if their p-value remained < 0.20. Variables excluded based on the univariable analyses were subsequently added one by one into the logistic regression model.

The final multivariable model was chosen based on Akaike Information Criterion and Bayesian Information Criterion. All four analyses were assessed for the following: linear relationships between the logit of the outcome and age using scatterplots; intercorrelation between independent variables, with variance inflation factors (VIF) > 5 used as the criterion for multicollinearity; and influential values, identified by examining standardized residuals, with values > 2 considered outliers.

Regression coefficients were expressed as odds ratios (OR) with 95 % confidence intervals (95 % CI) to indicate the strength of the association. The level of significance was set at 0.05. All analyses were conducted using the R statistical system version 3.5.2 (R Core Team 2021 Vienna, Austria).

## Results

The demographic data of the participants are presented in Table 1. Most of the physical therapists were female (*n* = 828, 67 %) and had worked as physical therapists for more than five years (*n* = 970, 79 %). Twenty-three percent (*n* = 275) of the physical therapists held a master’s degree or PhD, and 12 % (*n* = 147) were advanced clinical specialists. Most physical therapists used PROMs for pain (*n* = 885, 83 %), while the use of PROMs for disability (*n* = 297, 28 %), health-related (*n* = 152, 14 %), and psychosocial factors (*n* = 141, 13 %) were less common (Table 1).

**Table 1:** Demographic characteristics for all participants (*n* = 1237) and the number of those who reported using the specific PROMs in the groups: pain, disability, health-related and psychosocial factors.

	All physical therapists (*n* = 1237)	Use of PROMs pain (*n* = 885)	Use of PROMs health-related (*n* = 152)	Use of PROMs disability (*n* = 297)	Use of PROMS psychosocial (*n* = 141)
Female/male, n ( %)	828/409 (67/33)	613/271 (69/31)	111/41 (73/27)	205/91 (69/31)	97/43 (69/31)
Age, mean (SD)	44 (12)	44 (12)	47 (12)	45 (11)	47 (11)
*Education level,* n ( %) Bachelor’s degree Master’s degree or PhD	945 (77) 275 (23)	667 (76) 206 (24)	99 (66) 52 (34)	191 (65) 102 (35)	83 (60) 55 (40)
Advanced clinical specialist, n ( %)	147 (12)	111 (13)	36 (24)	57 (19)	31 (22)
Further education towards specialists, n ( %)	1047 (86)	766 (88)	140 (93)	268 (91)	121 (86)
*Current work line,* n ( %) Public health care centre Private health care centre Private clinic	583 (48) 137 (11) 499 (41)	415 (47) 97 (11) 367 (42)	59 (39) 12 (8) 81 (53)	123 (42) 27 (9) 145 (49)	72 (52) 10 (7) 57 (41)
*Years of working experience*, n ( %) <5 years 6–10 years 11–15 years 16–20 years >20 years	266 (21) 172 (14) 147 (12) 144 (12) 507 (41)	191 (22) 130 (15) 103 (12) 112 (13) 347 (39)	19 (13) 21 (14) 16 (11) 18 (12) 78 (51)	47 (16) 45 (15) 35 (12) 49 (17) 119 (40)	19 (14) 17 (12) 16 (11) 19 (14) 69 (49)
*How often meet patients with low back pain*, n ( %) Daily One to four times a week Less than once a week	720 (59) 442 (36) 67 (5)	535 (61) 324 (37) 25 (3)	96 (63) 49 (32) 7 (5)	174 (59) 111 (38) 11 (4)	79 (56) 53 (38) 8 (6)

PROMs, patient-reported outcome measures; SD, standard deviation.

### Association with the physical therapists' use of specific PROM groups

The results from the multivariable logistic analyses are presented below. Univariable analyses are presented in a Supplementary file.

#### PROMs for pain

Being a female (OR 2.57, 95 % CI 1.84, 3.59) and working in a private clinic (OR 1.83, 95 % CI 1.27, 2.67), compared to a public health care centre, were associated with the use of PROMs measuring pain (Table 2).

**Table 2:** Association between demographic variables and use of PROMs for pain among the participants (*n* = 885) based on a multiple logistic regression model.

	**OR (95 % CI)**	**Standard error**	**p**
*Sex* (Ref, Male) Female	2.57 (1.84, 3.59)	0.17	<0.001
*Current work line* (Ref, Public health care centre) Private health care centre Private clinic	0.94 (0.58, 1.56) 1.83 (1.27, 2.67)	0.25 0.19	0.79 <0.001

CI, confidence interval; OR, odds ratio; PROMs, patient-reported outcome measures.

#### PROMs for disability

Having a master’s degree or PhD (OR 1.85, 95 % CI: 1.28, 2.66), working in a private clinic (OR 1.39, 95 % CI: 1.01, 1.90), and having a work experience of 16–20 years (OR 2.02, 95 % CI: 1.22, 3.35) were associated to the use of PROMs for disability (Table 3).

**Table 3:** Association between demographic variables and use of PROMs for disability among the participants (*n* = 152) based on a multiple logistic regression model.

	**OR (95 % CI)**	**Standard error**	**p**
*Education level* (Ref, Bachelor’s degree) Master’s degree or PhD	1.85 (1.28, 2.66)	0.18	0.001
*Years of working experience* (Ref, <5 years) 6–10 years 11–15 years 16–20 years >20 years	1.42 (0.87, 2.30) 1.25 (0.73, 2.10) 2.02 (1.22, 3.35) 1.24 (0.82, 1.89)	0.34 0.36 0.35 0.27	0.16 0.41 0.006 0.32
*Specialist* (Ref, not specialist) Advanced clinical specialist	1.44 (0.90, 2.30)	0.24	0.13
*Current work line* (Ref, Public health care centre) Private health care centre Private clinic	0.89 (0.54, 1.43) 1.39 (1.01, 1.90)	0.33 0.19	0.64 0.04

CI, confidence interval; OR, odds ratio; PROMs, patient-reported outcome measures.

#### PROMs for health-related and psychosocial factors

Being a female, (OR 1.51, 95 % CI: 1.01, 2.29) and being an advanced clinical specialist (OR 2.09, 95 % CI: 1.29, 3.33) were associated with the use of PROMs for health-related factors (Table 4). To have a master's degree or PhD (OR 2.11, 95 % CI: 1.19, 3.65) was associated with using PROMs for psychosocial factors (Table 5).

**Table 4:** Association between demographic variables and use of PROMs for health-related factors among the participants (*n* = 297) based on a multiple logistic regression model.

	**OR (95 % CI)**	**Standard error**	**p**
*Sex* (Ref, Male) Female	1.51 (1.01, 2.29)	0.21	0.04
Age	1.02 (1.01, 1.04)	0.01	0.008
*Specialist* (Ref, not specialist) Advanced clinical specialist	2.09 (1.29, 3.33)	0.24	0.002
*Current work line* (Ref, Public health care centre) Private health care centre Private clinic	0.93 (0.46, 1.75) 1.43 (0.97, 2.14)	0.34 0.20	0.82 0.08

CI, confidence interval; OR, odds ratio; PROMs, patient-reported outcome measures.

**Table 5:** Association between demographic variables and use of PROMs for psychosocial factors among the participants (*n* = 141) based on a multiple logistic regression model.

	**OR (95 % CI)**	**Standard error**	**p**
Age	1.03 (1.01, 1.06)	0.01	0.005
*Education level* (Ref, Bachelors’ degree) Masters’ degree or PhD	2.11 (1.19, 3.65)	0.28	0.009

CI, confidence interval; OR, odds ratio; PROMs, patient-reported outcome measures.

## Discussion

Important factors associated with the physical therapists' use of PROMs for disability, health-related, and psychosocial factors were educational level (Master/PhD) or being an advanced clinical specialist. Our findings largely align with previous research on the overall use of PROMs, reporting that education and increased knowledge seem to be associated with a higher use.^28^^,^^40^, ^41^, ^42^ Copeland et al.^40^ surveyed physical therapists in New Zealand on their use of PROMs and reported that 40 % used PROMs, which was explained by having a master’s degree or education about PROMs.^40^ This was also the case among physical therapists in Saudi Arabia, where those with a higher education or an advanced specialty were more likely to use PROMs.^42^ Among Dutch physical therapists, increased knowledge and the use of an electronic journal system were associated with a higher likelihood of utilizing PROMs.^41^ In contrast, Brinkman et al.^61^ found that the physical therapists' use of PROMs was more closely linked to patient characteristics. They noted that physical therapists often select patients for PROMs based on their preferences.^61^

Unlike recent studies on factors affecting physical therapists' use of PROMs for managing LBP, we examined four groups of specific PROMs that measure various constructs. The low-to-very-low percentage of physical therapists who used PROMs for health-related (14 %) and psychosocial factors (13 %) was associated with being an advanced clinical specialist or having a higher educational level. This is somewhat consistent with the findings of Otero-Ketterer et al.^28^ who reported that only 14 % of surveyed physical therapists in Spain assessed psychosocial factors in clinical reasoning. Furthermore, only 50 % of these physical therapists used structured PROMs.^28^ The use of these PROMs was associated with working in a larger city, being in a private practice setting, or having received education about psychosocial factors.

Psychosocial factors are well-established predictors of persistent pain and disability in LBP.^62^ However, there is a low utilization of PROMs that assess these factors for patients with LBP, contrasting with recommendations found in the literature^11^^,^^12^ and clinical guidelines.^8^, ^9^, ^10^ Instead, in the clinical reasoning process, physical therapists' decision-making and treatment are reported to be influenced by the physical therapists' background, their perception of the patient, and their clinical experience.^6^^,^^41^ Physical therapists do not use PROMs to measure health-related and psychosocial factors, which may reflect their continued focus on biomechanical factors when treating LBP, despite recommendations advocating a psychosocial approach.^8^^,^^63^^,^^64^ Gardner et al.^64^ reviewed physical therapists' beliefs and attitudes in managing patients with LBP. They concluded that physical therapists tend to adopt a biomedical approach, focusing on tissue damage and selecting treatments based on this model which may overlook the influence of psychosocial factors.^64^ Another explanation might be that physical therapists are not confident or knowledgeable in delivering psychosocial interventions based on the outcomes of such PROMs.^65^ A review and meta-synthesis summarized that while physical therapists report a shift towards more bio-psychosocial and person-centred approaches, training interventions do not adequately help them feel confident in delivering all aspects.^65^ Furthermore, overall barriers to utilizing PROMs are due to several factors such as lack of time, competence, routine, and willingness to use them among patients and physical therapists.^27^ In addition, patients might not be motivated to complete or use PROMs due to time and reasons not measured in this trial. Future studies could therefore examine factors at the patient level such as their motivation and understanding of specific PROMs.

Our findings indicate that physical therapists with higher education levels are more likely to use specific PROMs. However, achieving a higher education does not necessarily mean to be informed about certain PROMs or follow recommended management strategies for patients with LBP. To further address the barriers to implementing specific PROMs, a transition strategy may be needed to enhance knowledge and change behaviours, ultimately increasing their usage.^66^ Educational meetings alone or combined with other interventions might improve professional practice, thus increasing the use of PROMs.^67^ Nevertheless, recent studies examining educational interventions involving PROMs have yielded varied results. One recent study explored the implementation of a guideline-endorsed treatment strategy for LBP based on the biopsychosocial model.^68^ The physical therapists' biopsychosocial orientation increased after implementation but decreased over time, highlighting the need to monitor changes.^69^ In addition, a study conducted among physical therapists in the Netherlands reported that the use of PROMs was not as high as expected following an implementation project.^70^ To enhance the use of PROMs for health-related and psychosocial factors, it may be essential to implement educational interventions that increase knowledge of a biopsychosocial approach to LBP, before expecting physical therapists to use such PROMs.

The strength of the present study is that we included a large group of physical therapists (*n* = 1237) working in primary health care in Sweden. Even so, we cannot rule out the possibility that the results might not represent all physical therapists working in primary care in Sweden as only 25 % of all surveyed answered. A possible limitation is the recall bias, which may have caused physical therapists to over- or underestimate their use of PROMs when answering the survey, potentially impacting the results. A further limitation is that we did not control for possible confounding factors in the multivariable analyses but still included demographic factors such as age and sex in the univariable analyses. However, we cannot rule out the risk of unmeasured confounding impacting our results.

The decision to classify the included PROMs into four groups can be debated. This was informed by findings from a previously published survey study on the same data, demonstrating a diverse use of various PROMs where few physical therapists (<15 %) used PROMs for health-related and psychosocial factors.^26^ We also chose to include the shorter PROMs used to stratify patients to risk for long-term pain and disability in the PROM group for psychosocial factors as they mainly comprise questions on such factors.

In hindsight, and to better understand what factors are associated with the physical therapists’ use of PROMs for managing patients with LBP, it would have been valuable to include questions about physical therapists’ attitudes, beliefs, and motivation to use them as this has been reported to be important.^64^ Other factors, such as patient expectations, may also influence the physical therapists’ use of certain PROMs in the clinical reasoning process, to align with the patient’s preference.

## Conclusion

Few physical therapists use PROMs to assess health-related and psychosocial factors. Those with higher educational levels or advanced clinical specialists are more likely to incorporate such PROMs when managing patients suffering from LBP. Future studies need to examine educational interventions to enhance the understanding of the importance of specific PROMs in clinical reasoning from a biopsychosocial perspective.

## Declaration of competing interest

None to declare.

## Appendix Supplementary materials

Image, application 1
