# Supplementary material for: What factors are associated with physical therapists’ use of patient-reported outcome measures in managing patients with low back pain in primary health care in Sweden?
Source: Braz J Phys Ther. 2025 Aug 12;29(6):101250. doi: 10.1016/j.bjpt.2025.101250 (PMC12361981; doi:10.1016/j.bjpt.2025.101250)
Supplement: Supplementary file 1 [file mmc1.docx]

**Supplementary file**

| **Table 1.** Association between demographic variables and the physical therapists’ use of PROMs for pain based on *univariable* logistic regression analyses. | | | |
| --- | --- | --- | --- |
|  | OR (95% CI) | Standard error | p-value |
| *Sex* (Ref, Male)  Female | 2.42 (1.75,3.35) | 0.17 | <0.001 |
| Age | 1.00 (0.99,1.02) | 0.01 | 0.75 |
| *Education level* (Ref, Bachelor’s degree)  Master’s degree or PhD | 1.38 (0.92,2.12) | 0.21 | 0.13 |
| *Further education* (Ref, no further education)  Further education towards specialist | 0.84 (0.49,1.37) | 0.26 | 0.5 |
| *Years of working experience* (Ref, <5 years)  6-10 years  11-15 years  16-20 years  >20 years | 1.28 (0.74,2.22)  1.06 (0.75,2.22)  1.76 (0.96,3.40)  1.12 (0.74,1.68) | 0.28  0.28  0.32  0.21 | 0.34  0.85  0.08  0.59 |
| *Specialist* (Ref, not specialist)  Advanced clinical specialist | 1.47 (0.77,2.64) | 0.28 | 0.17 |
| *Current work line* (Ref, Public health care center)  Private health care center  Private clinic | 0.88 (0.55,1.45)  1.60 (1.12,2.31) | 0.25  0.18 | 0.61  0.01 |
| *How often meet patients with low back pain* (Ref, daily)  One to four times a week  Less than once a week | 1.18 (0.84,1.67)  0.90 (0.38,2.46) | 0.17  0.47 | 0.34  0.81 |
| PROM patient reported outcome measure, OR, odds ratio; CI, confidence interval. | | | |

| **Table 2.** Association between demographic variables and the physical therapists’ use of PROMs for health-related factors based on *univariable* logistic regression analyses. | | | |
| --- | --- | --- | --- |
|  | OR (95% CI) | Standard error | p-value |
| *Sex* (Ref, Male)  Female | 1.47 (1.01,2.17) | 0.12 | 0.05 |
| Age | 1.03 (1.02,1.05) | 0.01 | <0.001 |
| *Education level* (Ref, Bachelor’s degree)  Master’s degree or PhD | 2.01 (1.38,2.90) | 0.19 | <0.001 |
| *Further education* (Ref, no further education)  Further education towards specialist | 1.92 (1.05,3.86) | 0.33 | 0.04 |
| *Years of working experience* (Ref, <5 years)  6-10 years  11-15 years  16-20 years  >20 years | 1.81 (0.94,3.52)  1.65 (0.81,3.34)  1.88 (0.94,3.74)  2.65 (1.59,4.63) | 0.34  0.36  0.35  0.27 | 0.08  0.16  0.07  <0.001 |
| *Specialist* (Ref, not specialist)  Advanced clinical specialist | 2.82 (1.81,4.32) | 0.22 | <0.001 |
| *Current work line* (Ref, Public health care center)  Private health care center  Private clinic | 0.83 (0.41,1.54)  1.85 (1.29,2.68) | 0.33  0.19 | 0.57  0.001 |
| *How often meet patients with low back pain* (Ref, daily)  One to four times a week  Less than once a week | 0.84 (0.58,1.21)  1.66 (0.65,3.77) | 0.19  0.44 | 0.36  0.25 |
| PROM patient reported outcome measure, OR, odds ratio; CI, confidence interval. | | | |

| **Table 3.** Association between demographic variables and the physical therapists’ use of PROMs for disability based on *univariable* logistic regression analyses. | | | |
| --- | --- | --- | --- |
|  | OR (95% CI) | Standard error | p-value |
| *Sex* (Ref, Male)  Female | 1.23 (0.92,1.64) | 0.15 | 0.17 |
| Age | 1.01 (1.00,1.03) | 0.01 | 0.02 |
| *Education level* (Ref, Bachelor’s degree)  Master’s degree or PhD | 2.41 (1.78,3.27) | 0.16 | <0.001 |
| *Further education* (Ref, no further education)  Further education towards specialist | 1.51 (0.98,2.40) | 0.23 | 0.07 |
| *Years of working experience* (Ref, <5 years)  6-10 years  11-15 years  16-20 years  >20 years | 1.67 (1.04,2.68)  1.54 (0.93,2.54)  2.58 (1.59,4.18)  1.65 (1.13,2.44) | 0.24  0.26  0.25  0.20 | 0.03  0.09  <0.001  0.01 |
| *Specialist* (Ref, not specialist)  Advanced clinical specialist | 2.43 (1.66,3.57) | 0.20 | <0.001 |
| *Current work line* (Ref, Public health care center)  Private health care center  Private clinic | 0.87 (0.53,1.38)  1.66 (1.25,2.22) | 0.24  0.15 | 0.57  <0.001 |
| *How often meet patients with low back pain* (Ref, daily)  One to four times a week  Less than once a week | 1.12 (0.84,1.48)  1.64 (0.74,3.50) | 0.14  0.39 | 0.45  0.21 |
| PROM patient reported outcome measure, OR, odds ratio; CI, confidence interval. | | | |

| **Table 4.** Association between demographic variables and the physical therapists’ use of PROMs for psycho-social factors based on *univariable* logistic regression analyses. | | | |
| --- | --- | --- | --- |
|  | OR (95% CI) | Standard error | p-value |
| *Sex* (Ref, Male)  Female | 1.20 (0.82,1.77) | 0.20 | 0.36 |
| Age | 1.03 (1.01,1.04) | 0.01 | 0.001 |
| *Education level* (Ref, Bachelor’s degree)  Master’s degree or PhD | 2.59 (1.77,3.77) | 0.19 | <0.001 |
| *Further education* (Ref, no further education)  Further education towards specialist | 0.86 (0.52,1.49) | 0.27 | 0.57 |
| *Years of working experience* (Ref, <5 years)  6-10 years  11-15 years  16-20 years  >20 years | 1.43 (0.71,2.86)  1.65 (0.81,3.34)  2.06 (1.04,4.07)  2.33 (1.39,4.09) | 0.35  0.36  0.35  0.27 | 0.31  0.16  0.04  0.002 |
| *Specialist* (Ref, not specialist)  Advanced clinical specialist | 2.49 (1.57,3.89) | 0.23 | <0.001 |
| *Current work line* (Ref, Public health care center)  Private health care center  Private clinic | 0.54 (0.25,1.03)  0.97 (0.66,1.40) | 0.35  0.19 | 0.08  0.86 |
| *How often meet patients with low back pain* (Ref, daily)  One to four times a week  Less than once a week | 1.16 (0.80,1.68)  2.70 (1.09,6.08) | 0.19  0.43 | 0.43  0.02 |
| PROM patient reported outcome measure, OR, odds ratio; CI, confidence interval. | | | |
